# Large, segmental, circular vascular malformation of the small intestine (in a female toddler with hematochezia): unusual presentation in a child

**DOI:** 10.1186/1471-2431-14-55

**Published:** 2014-02-26

**Authors:** Peter I Kalmar, Thomas Petnehazy, Ulrike Wießpeiner, Meinrad Beer, Almuthe C Hauer, Holger Till, Michael Riccabona

**Affiliations:** 1 Department of Radiology, Division of Pediatric Radiology, Medical University of Graz, Graz, Austria; 2 Department of Pediatric and Adolescence Surgery, Division of Pediatric and Adolescence Surgery, Medical University of Graz, Graz, Austria; 3 Department of Diagnostic and Interventional Radiology, University Hospital of Ulm, Ulm, Germany; 4 Department of Pediatrics and Adolescence Medicine, Medical University of Graz, Graz, Austria

**Keywords:** Abdominal mass, Bowel obstruction, Gastrointestinal bleeding, Children (Child, Pediatric)

## Abstract

**Background:**

Failure to thrive and hematochezia in children may be alarm signs warranting endoscopy. In contrast, vascular malformations of the small intestine are uncommon in this age group. We report on a female toddler in whom various imaging techniques revealed an unusually large segmental vascular malformation of the ileum as the cause of the child’s main clinical symptoms.

**Case presentation:**

A 19 months old girl presented with severe anemia (Hb 3 mmol/l), failure to thrive and chronic diarrhea. Diagnostics for intestinal blood loss and pathogens were negative. The child had duodenoscopy, also for histological diagnosis of celiac disease, with negative results. A dietary protocol was suggestive for inadequate iron intake and she was supplemented. After symptomless four-months the child presented again, now with mild abdominal pain and, for the first time, hematochezia. An orienting abdominal ultrasound (US) study showed a suspicious tumorous bowel condition. A subsequent detailed abdominal US supplemented by a saline enema during investigation (i.e., “hydrocolon”, to improve outlining of the formation’s localization) revealed a large circumferential cystiform vascular mass of the ileum causing segmental ileal obstruction.

Complementing preoperative abdominal hydro-MRI, planned based on the findings of the US study, confirmed the suspected vascular malformation of the ileum and exquisitely outlined the extent, location and anatomy.

The patient was successfully operated laparoscopically, the affected ileum segment with the mass was completely removed as proven by histology, and the child recovered well.

**Conclusions:**

The huge segmental vascular malformation of the distal ileum described here is an extreme rarity in young children. Although the reported child’s presenting symptoms malabsorption and malnutrition could have been responsible for its severe anemia, this was obviously caused by blood losses from the ileal vascular malformation. It was due to incipient abdominal pain rather than hematochezia that abdominal US was performed and proved crucial for correctly diagnosing this rare malformation. Even in this extensive case detailed imaging work-up including adapted MRI added all information necessary for minimal invasive laparoscopic en bloc resection.

## Background

Both failure to thrive with chronic diarrhea as well as hematochezia are clinical alarm signs in children potentially warranting endoscopy. And while hematochezia has a great variety of causes, vascular malformations of the small intestine - as one of them - are uncommon in this age group. In particular, there are only very few reports on this disease in children younger than two years [1-4].

Although infants and children suffering from intestinal vascular malformations may present with symptoms such as abdominal pain, gastrointestinal bleeding, diarrhea or vomiting, some cases are asymptomatic and thus remain hidden from the diagnostician’s eye for quite some time. On the other hand, very few cases may lay a false trail for the clinician because of unaccompanied anemia [5]. Early imaging using optimal technique is crucial for diagnosing these conditions, in the pediatric age group usually best performed by detailed ultrasound (US), potentially supplemented by MRI.

This is demonstrated by our report of a 19 months old female toddler who presented first with failure to thrive and signs of malabsorption and much later with abdominal pain and hematochezia as the clinical symptoms leading to the diagnosis of an unusual large, segmental, circumferential, low-flow vascular malformation of the ileum.

## Case presentation

A 19 months old girl presented with severe anemia (Hb 3 mmol/l). Her parents had noticed her failure to thrive and chronic diarrhea. There was no visible blood in her stools, and markers for intestinal blood loss as well as assays for intestinal pathogens were negative. Also because of inconclusive celiac serology the child underwent duodenoscopy with normal macroscopic and histologic findings. Since a detailed dietary protocol kept by her parents pointed towards (iron) malnutrition, she was adequately supplemented.

After a symptomless four-month interval the child presented again. It was only now that her parents had noted hematochezia and also suspected occasional abdominal pain. Physical examination showed no abnormalities, in particular no abdominal mass on palpation. Laboratory tests were normal apart from mild neutropenia and slighty elevated d-dimer values. However, guaiac fecal occult blood test (HaemOccult®, Beckman Coulter Inc., Brea, CA/USA) was positive.

**Figure 1:**
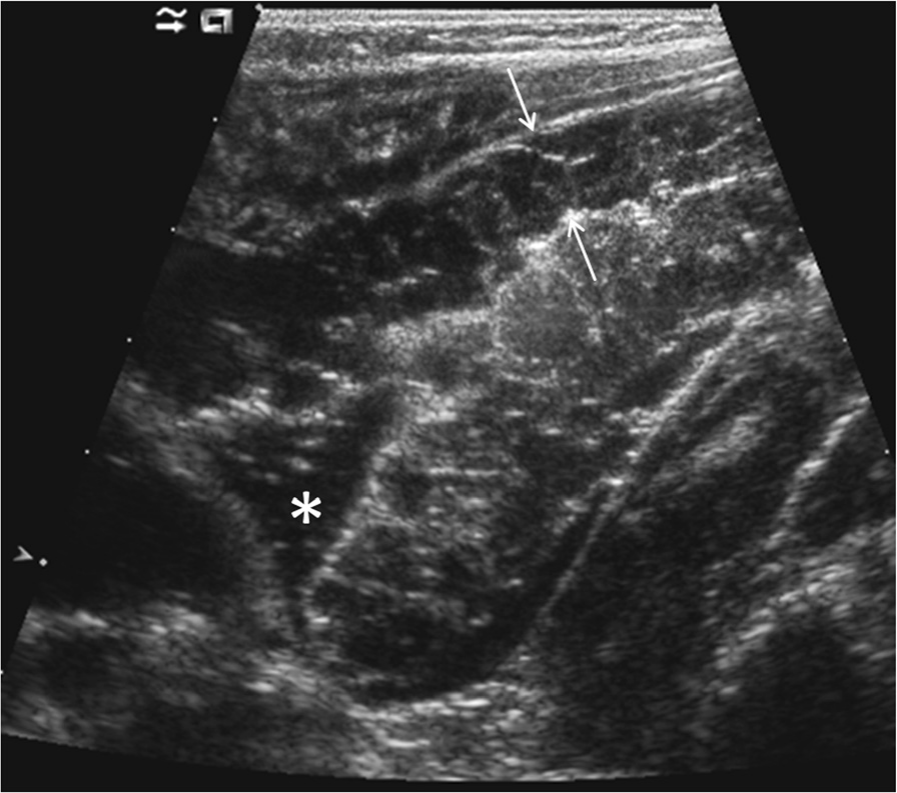
**Hydrocolon ultrasound image taken after gentle administration of 500 ml saline enema.** Circular lobulated infiltration of the ileum wall (arrows), with multiple cystiform-tubular anechoic, partially confluent areas (consistent with venous structures) causing a concentric stenosis. The enema fluid entering the stenotic section is well depicted (*).

Abdominal sonography was performed and revealed a 10 to 15 cm long circular lobulated infiltration of the ileum wall, the aboral end almost extending to Bauhin’s valve (Figure 1). The lesion’s transmural extension was 1.5 cm and caused a concentric luminal stenosis. Locoregional small amounts of ascites were present. No enlarged lymph nodules were visible. Colour Doppler sonography revealed venous flow patterns in many of the tubular-unechoic multifocal structures in and around the lesion. On duplex Doppler sonography, the larger vessels presented predominantly low-pressure and venous low-flow characteristics, without major feeding or draining vessels; some vessels appeared to be thrombosed.

In order to obtain information on the lesion’s intraluminal or stenosing aspects as well as its exact location an enema consisting of 500 ml saline was gently administered during the US. Filling of the entire colon was prompt and uneventful and confirmed the ileal location of the mass. We then used high frequency linear probes to precisely document the enema’s remarkably prolonged passage which was due to high grade stenosis of the affected small bowel segments. However, clear definition of its relation to Bauhin’s valve was impossible by distension only and without the additional means of analgo-sedation.

**Figure 2:**
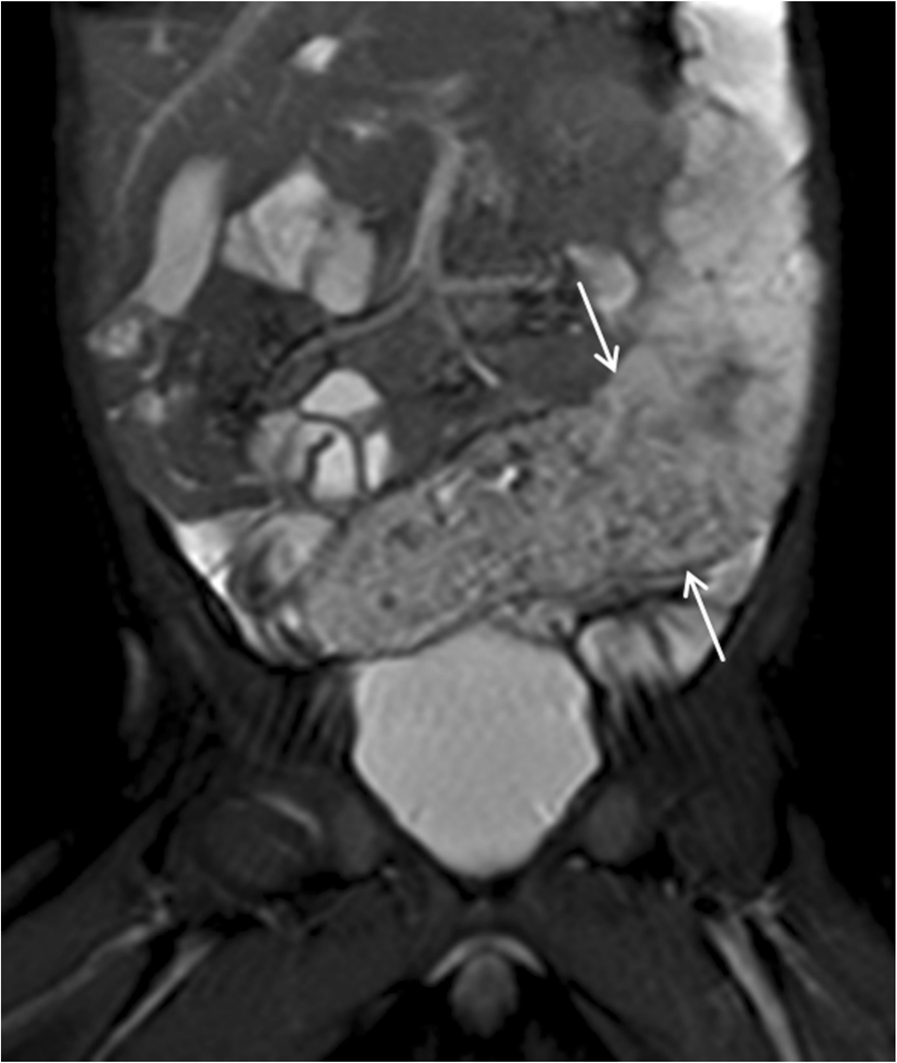
MRI: coronal TruFISP image demonstrating the vascular malformation (arrows).

Abdominal Hydro-MRI based on the US results was subsequently performed to further visualize extent, location, and anatomic details. The circular infiltration of the terminal ileum (Figure 2) was thus confirmed. The lesion exhibited delayed, almost homogenous enhancement after intravenous administration of the contrast agent. Additionally, no large feeding or draining vessels were depicted on MR-angiography, performed in the early phases of contrast agent administration. Small amounts of ascites, but no enlarged lymph nodules nor large feeding vessels were found.

**Figure 3:**
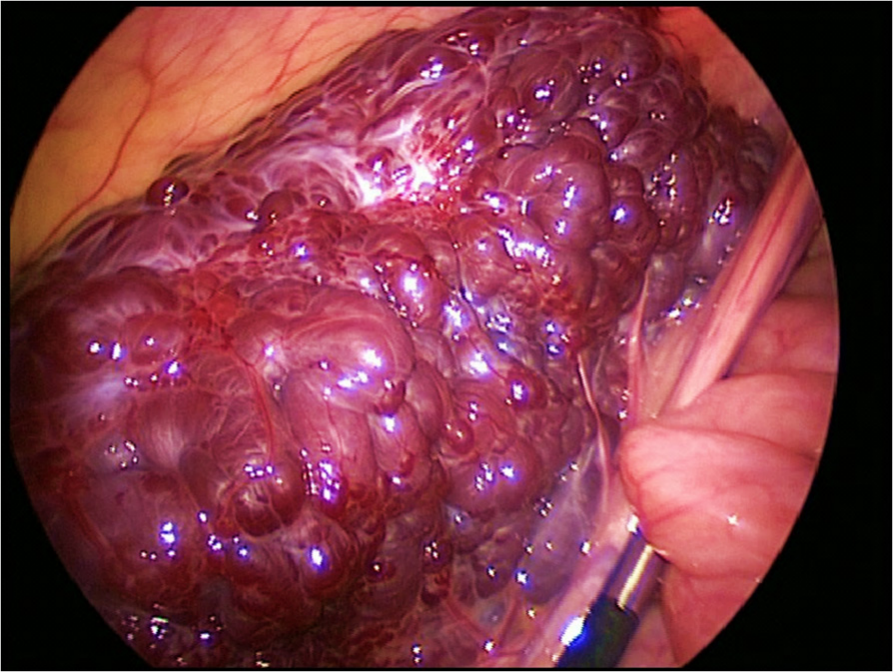
**Intraoperative photograph.** The purple bacciform vascular malformation is completely encircling the affected terminal ileum.

Due to worsening of clinical symptoms and remaining uncertainty as to the potentially malignant nature of the lesions an interdisciplinary committee decided on diagnostic laparoscopy. A three-port-laparoscopy (3-5 mm) was performed and revealed a purple bacciform vascular malformation of the mid-ileum completely encircling the affected intestinal segment (Figure 3). Since Bauhin’s valve was not affected as primarily indicated by the imaging studies, the pediatric surgeons decided to remove the 12 cm long malformation en bloc. After a median subumbilical minilaparotomy the lesion was externalized via an Alexis® Wound Retractor, resected and finally an ileal end-to-end anastomosis was performed. Macroscopically, the resected tissue showed multiple, partially hemorrhagic cystic structures smaller than 6 mm which were bulging the focally livid serosa. Histology defined the lesion as a vascular malformation due to innumerable partially thrombosed small blood vessels throughout the bowel wall with alternating vessel diameter and wall thickness. There were no signs of malignancy; in sano resection had been achieved.

The girl recovered well from the procedure and was able to leave the hospital in good general condition after one week. Early follow-up US revealed no residual or recurrent tumorous tissue.

## Conclusions

To our knowledge, there are worldwide only very few reports on vascular malformations of the small intestine in small children, mostly not larger than 3 cm [3-6]. Our case demonstrates a very extensive case of this rare entity with the mass occupying almost one quarter of the intraperitoneal cavity.

We assume intestinal blood loss from the vascular malformation to be the main cause for the child’s anemia. However, at initial presentation her leading symptoms, i.e. failure to thrive and malabsorption were believed to be the reason for her clinical condition, and thus - rather than imaging techniques - the more invasive endoscopy was chosen. Diagnosis was made only after the second admission when the diagnostic work-up included ultrasound because of incipient abdominal pain. It is of note that, again, clinical examination of the child’s abdomen proved to be normal as has been shown to be typical for bowel vascular malformations [7]. And it was only then that the child presented with bloody stools.

The consecutive thorough abdominal US study then detected the lesion in the wall of the distal ileum. Modern US techniques and approaches using high spatial resolution including assessment of low flow characteristics by sensitive Doppler and bowel distention by enema during the investigation enabled a prompt and reliable diagnosis. In equivocal cases one could additionally think about using potentially supported by extended view US, 3DUS or even contrast-enhanced US. Hydro-MRI planned according to the US results (i.e. MRI with rectal saline enema to fill and distend the colon and potentially the distal ileum) provided the definitive diagnosis of a vascular malformation (due to late and diffuse contrast enhancement) of the ileum, which was confirmed histologically. It furthermore helped to outline preoperatively relevant information such as location, length, anatomical details and the absence of large feeding or draining vessels which might have caused surgical complications.

The decision to perform surgery was made because the girl suffered from gastrointestinal bleeding (hematochezia), which required prompt diagnosis and therapy. Although the diagnostic workup immediately revealed a vascular anomaly of the distal ileum, the exact differentiation between a hemangioma and a complex vascular malformation could not be made. Thus medical treatment with propranolol or corticosteroid did not seem justified. Furthermore the MRI could not delineate the exact position or its extent to initiate sclerotherapy or embolization which would also have endangered the vitality of the affected bowel segment. In contrast, laparoscopy identified the affected segment of small bowel as well as the macroscopic borders of the lesion and thus lead to definite surgical therapy. After delivery through a minimally extended umbilical port sire the lesion could be resected completely, the pathology report revealed that the margins of resection were free of disease. Since then, the postoperative course of more than two years has been uneventful.

In conclusion, this case shows that common symptoms may be present even in an extremely rare pathology. Furthermore, it demonstrates that even early invasive diagnostics may still require additional less- or non-invasive methods to enable the correct diagnosis; maybe an initial US study can be considered compulsory before going for more invasive studies.

### Consent

Written informed consent was obtained from the patient for publication of this Case report and any accompanying images. A copy of the written consent is available for review by the Editor of this journal.

## Competing interest

The authors declare that they have no competing interests.

## Authors’ contributions

All authors 1) have made substantial contributions to conception and design, or acquisition of data, or analysis and interpretation of data 2) have been involved in drafting the manuscript or revising it critically for important intellectual content and 3) have given final approval of the version to be published.

## Pre-publication history

The pre-publication history for this paper can be accessed here:

http://www.biomedcentral.com/1471-2431/14/55/prepub
